# Circulation and Molecular Epidemiology of Enteroviruses in Paralyzed, Immunodeficient and Healthy Individuals in Tunisia, a Country with a Polio-Free Status for Decades

**DOI:** 10.3390/v13030380

**Published:** 2021-02-27

**Authors:** Anissa Chouikha, Dorra Rezig, Nadia Driss, Ichrak Abdelkhalek, Ahlem Ben Yahia, Henda Touzi, Zina Meddeb, Essia Ben Farhat, Mahrez Yahyaoui, Henda Triki

**Affiliations:** 1 Laboratory of Clinical Virology, WHO Reference Laboratory for Poliomyelitis and Measles in the Eastern Mediterranean Region, Pasteur Institute of Tunis, University Tunis El Manar (UTM), Tunis 1068, Tunisia; dorra.rezig@issbat.utm.tn (D.R.); nadiochka1982@yahoo.fr (N.D.); ichrak.abdelkhalek@yahoo.com (I.A.); aouiahlem@yahoo.fr (A.B.Y.); touzihenda@yahoo.fr (H.T.); zinamedeb@gmail.com (Z.M.); henda.triki@pasteur.rns.tn (H.T.); 2 Research Laboratory, LR20IPT02, Pasteur Institute of Tunis, Tunis 1006, Tunisia; 3 National Program of Immunization Basic Health Care Division, Ministry of Health Tunis, Tunis 1006, Tunisia; essia.hmida@gmail.com (E.B.F.); mahrezyahyaoui@gmail.com (M.Y.)

**Keywords:** *Picornaviridae*, poliovirus, nonpolio enterovirus, primary immunodeficiencies, VDPV, acute flaccid paralysis, epidemiology

## Abstract

This report is an overview of enterovirus (EV) detection in Tunisian polio-suspected paralytic cases (acute flaccid paralysis (AFP) cases), healthy contacts and patients with primary immunodeficiencies (PID) during an 11-year period. A total of 2735 clinical samples were analyzed for EV isolation and type identification, according to the recommended protocols of the World Health Organization. Three poliovirus (PV) serotypes and 28 different nonpolio enteroviruses (NPEVs) were detected. The NPEV detection rate was 4.3%, 2.8% and 12.4% in AFP cases, healthy contacts and PID patients, respectively. The predominant species was EV-B, and the circulation of viruses from species EV-A was noted since 2011. All PVs detected were of Sabin origin. The PV detection rate was higher in PID patients compared to AFP cases and contacts (6.8%, 1.5% and 1.3% respectively). PV2 was not detected since 2015. Using nucleotide sequencing of the entire VP1 region, 61 strains were characterized as Sabin-like. Among them, six strains of types 1 and 3 PV were identified as pre-vaccine-derived polioviruses (VDPVs). Five type 2 PV, four strains belonging to type 1 PV and two strains belonging to type 3 PV, were classified as iVDPVs. The data presented provide a comprehensive picture of EVs circulating in Tunisia over an 11-year period, reveal changes in their epidemiology as compared to previous studies and highlight the need to set up a warning system to avoid unnoticed PVs.

## 1. Introduction

The genus *Enterovirus* belongs to the *Picornaviridae* family of small nonenveloped viruses with icosahedral symmetry and a single-stranded positive-sense RNA genome of approximately 7500 nucleotides [1]. This genus is divided into 15 species, 7 of which contain human pathogenic viruses, namely the species Enterovirus Α-D, including polioviruses, echoviruses and coxsackieviruses as well as Rhinovirus A-C [2]. Enteric enteroviruses (EVs) are transmitted fecal-orally and replicate primarily in the gastrointestinal tract from where they can sporadically disseminate and cause infection in several other organs. EVs have been associated with a wide range of illnesses, including aseptic meningitidis, myocarditis, newborn sepsis, conjunctivitis, hepatitis and severe flaccid paralysis [3]. 

The *Enterovirus* genus was first recognized by the discovery of polioviruses (PVs) in 1953. PVs are the leading cause of acute flaccid paralysis (AFP) in infants and young children. Since 1988, wild PVs have been targeted by a global eradication program based on the mass vaccination of the human population and attentive surveillance of circulating PVs, especially in AFP cases and their contacts. The prevalence of poliomyelitis decreased dramatically with the widespread use of polio vaccines, especially the oral poliovirus vaccine (OPV), developed by Sabin and containing live attenuated strains from the three PV serotypes. The endemic circulation of wild PVs is now interrupted in most countries, and we are probably very close to eradication. Indeed, the eradication of type 2 wild poliovirus (WPV) was certified in 2015, and the transition from trivalent OPV to bivalent OPV was implemented in April 2016. The last type 3 WPV was detected in Nigeria in November 2012, and it was declared eradicated in 2019 [4,5,6]. However, a big concern with the use of OPV is the emergence of vaccine-derived polioviruses (VDPVs) [7,8], defined as viruses that have genetically evolved from the parental Sabin strains during replication in the human gut and person-to-person transmission. VDPVs currently constitute a big challenge to reach the final success of the program, given their potential to cause paralysis and/or persistent circulation in human communities. They are identified based on their degree of genetic divergence from the parental Sabin strain: VDPVs of types 1 and 3 have >1% and VDPVs of type 2 have >0.6% genetic divergence. VDPVs were classified into different categories, the most important are circulating VDPVs (cVDPVs), showing evidence of person-to-person transmission, and immunodeficient VDPVs (iVDPVs), isolated from patients with primary immunodeficiencies (PIDs). Currently, immunodeficient patients exposed to OPV may harbor the vaccine virus for many months or years [9] and constitute a potential reservoir for potentially neurovirulent PVs after the eradication of WPVs. With currently no licensed and effective antiviral treatment to stop excretion in these patients, efforts to detect chronic excretors are needed to better assess the risk posed by iVDPVs.

Unlike PVs, and with the lack of specific vaccines, nonpolio enteroviruses (NPEVs) remain widespread globally. They cause a wide variety of clinical diseases, especially in children. Most infections are asymptomatic or mild, but serious illnesses may occur, such as encephalitis, meningitis, paralysis, pericarditis, myocarditis and sepsis. Severe forms of EV infection, especially with neurological presentation (meningitis, encephalitis, paralysis), were frequently reported in immunodeficient patients [10,11,12]. Several developed countries have established national EV surveillance systems that collect laboratory data on circulating EVs. Such surveillance systems might be used to determine patterns of circulation for individual virus types, interpret trends in enteroviral diseases, assist with the recognition of outbreaks and guide the development of new diagnostic tests and therapies [13,14]. 

In Tunisia, the national immunization program has used OPV since 1979 for 90% of Tunisian children who attend government vaccination centers; the remaining 10% are vaccinated in the private sector and receive the inactivated polio vaccine (IPV). The national immunization program includes 7 doses of polio vaccine delivered at 2, 3, 6 and 18 months of age, with three supplemental doses in school-aged children at 6, 12 and 18 years of age. High coverage rates with polio vaccines have been maintained for years, and wild polioviruses were not detected since 1994. Tunisia is now progressively replacing OPV with IPV: one dose of IPV was introduced in the national immunization program in 2015 and a second dose in 2016. Additionally, in line with the global program for type 2 PV containment, trivalent OPV was replaced by bivalent OPV containing Sabin1 and Sabin3 only. The coverage rates with polio vaccines were maintained over 90% since the early 1990s, and national immunization days were conducted from 1995 to 1997. Attentive virological investigation of all detected polio-suspected cases (AFP cases) was started in 1991. The Laboratory of Clinical Virology in Pasteur institute of Tunis serves as the National Reference Laboratory and, accordingly, all stool samples collected in polio-suspected cases and their healthy contacts are forwarded for virological investigation. The laboratory also receives stools for EV detection in immunodeficient patients. The last paralytic case due to a WPV occurred in 1992, and the last wild isolate was detected in 1994. Previous reports showed that, since 1995 and up to 2006, all PV isolates detected through the national surveillance program for poliomyelitis were vaccine-related [15,16]. This surveillance activity also detects several NPEVs, their epidemiological patterns from 1992 to 2003 were previously described [15] with more than 20 different circulating serotypes, most of them belonging to EV-B species.

This paper reports the results of EV detection in Tunisian polio-suspected paralytic cases, healthy contacts and PID patients during an 11-year period starting from 2007. The aim is to describe the trends in the epidemiology of these pathogens and to highlight key findings that may have implications on the current late steps of the poliomyelitis eradication program.

## 2. Material and Methods

### 2.1. Studied Population and Sampling

A total of 2735 stool samples, collected during an 11-year period between January 2007 and December 2017, were investigated. Samples were collected from 607 polio-suspected paralytic cases (*n* = 1296, most of the cases had 2 stools collected at 24–48 h intervals), 825 healthy individuals in their contact (*n* = 825, 1 sample from each individual) and 290 collected in patients with PIDs (*n* = 611, 1 to 15 samples per patient). The number of tested samples ranged from 153 to 329 per year. Table 1 describes the studied population over the 11 years. All samples were collected by the polio surveillance teams in the different districts of the country. They were collected in sterile containers that were immediately stored at +4 °C and transported within 24–48 h to the Laboratory of Clinical Virology in Pasteur institute. Upon reception, the samples were kept refrigerated until preparation of stool suspensions, which was systematically performed within 24 h. The original samples were then stored frozen at −20 °C for longer conservation. Accuracy of the laboratory results is ensured through attentive adherence to WHO standard protocols, participation in annual external quality controls and annual accreditation of the laboratory by WHO experts.

### 2.2. Enterovirus Detection from Stool Samples 

Virus detection was performed by specimen inoculation on cell culture, according to the WHO standard protocols for PV surveillance. Stool extracts were prepared and inoculated onto WHO-recommended cell lines: RD (human rhabdomyosarcoma), L20B (transgenic mouse cell line expressing the gene of the human cellular receptor for PV) and HEp-2c (human larynx epidermoid carcinoma). The cultures were incubated at 37 °C and the cytopathic effect (CPE) was monitored daily, for ten days, by microscopic examination. Samples showing CPE in the L20B tubes were frozen and thawed for passage onto tubes with fresh RD monolayers. If the RD tube was positive, possible PV was reported in the corresponding L20B tube. On the other hand, any positive tube of RD was passed into a tube with fresh monolayer culture of L20B cells. If this L20B tube was positive, the L20B cell isolate was cross-passaged on a tube with fresh monolayer culture of RD cells. Any sample showing CPE in RD, from cross-passaged L20B isolate was considered as “suspected poliovirus.” Any culture negative in L20B but positive in RD and/or HEp-2c was classified as “nonpolio enterovirus.”

### 2.3. Identification of Viral Isolates

#### 2.3.1. Typing of Nonpolio enteroviruses 

NPEVs were first identified by the presence of a cytopathic effect on RD and/or HEp2-c cell lines with negative culture results on L20B cell lines. NPEVs were typed by sequencing part of the VP1 capsid protein gene that is the gold standard for EV typing [17]. Viral RNA extraction, reverse transcription and PCR amplification were conducted using a previously described “in-house” protocol [18] and the primers published by Oberste et al. [19]. PCR products were purified using the QIAquick Gel Extraction kit (QIAGEN, GmbH, Germany) and sequenced in both directions using an automated DNA sequencer (Applied Biosystem 3130) and the ABI BigDye Terminator cycle sequencing kit (Applied Biosystem, United States). Sequence data were analyzed using MEGA software version 7 [20]. The type was determined, as recommended [21], by comparing the obtained 356bp sequence to the sequences of the same region published in the GenBank database. EV types are defined based on a >75% nucleotide and >85% amino acid sequence identity to a prototype strain [22], although some isolates within a type have drifted slightly beyond these thresholds [23].

#### 2.3.2. Typing of Polioviruses and Identification of VDPVs

PVs were identified first by the presence of a cytopathic effect on L20B cell lines. They were then typed using a WHO standard real-time reverse transcription-polymerase chain reaction (RT-PCR) assay using pan-poliovirus- and serotype-specific primers. Confirmed PVs were further characterized as Sabin or wild strains by real-time RT-PCR using Sabin-like type-1-, 2- and 3-specific primers. All isolates were then analyzed by sequencing of the entire VP1 genomic region and the sequences were compared with those of Sabin-like strains. VDPVs were identified based on their degree of genetic divergence from the parental Sabin strain: VDPVs of types 1 and 3 have >1% (>10 nucleotide changes) and VDPVs of type 2 have >0.6% (>6 nucleotide changes) genetic divergence in the complete genomic region. If the nucleotide sequence variations of types 1 and 3 were between 6 and 9, they were judged as high-mutant strains (pre-VDPV) [24]. Mixed bases found were counted as nucleotide changes. Any PV isolate with any nucleotide difference from Sabin less than the number that met the definition of VDPV was identified as Sabin-like.

## 3. Results

Out of the 2735 stool samples investigated, EVs were detected in 227 (8.3%) samples, corresponding to 103 individuals; the remaining samples showed no cytopathic effect on any of the three cell lines used. 

### 3.1. Nonpolio Enterovirus Detection

**Figure 1:**
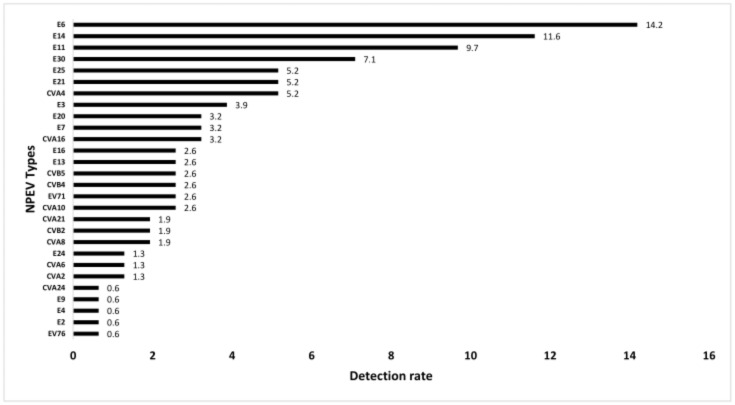
Detection rates of NPEV types.

Out of the 2735 stool samples tested, 155 (collected from 73 individuals) showed a CPE on RD and/or HEp2-c cell lines with no CPE on L20Bs and were positive for NPEVs: 76 from 13 patients with PIDs, 56 from 25 AFP cases and 23 from 23 healthy contacts. The NPEV detection rate was higher in patients with PIDs: 12.4% (76 positive out of 614 specimens) vs. 4.3% (56 positive out of 1296 specimens) and 2.8% (23 positive out of 825 specimens) in AFP cases and contacts, respectively. The results of the NPEV typing, based on partial VP1 sequencing, are shown in Table 2 and Figure 1. The Figure 1 shows the detection rates of NPEV types identified in this study, and the four most frequently observed NPEV types were E6, E14, E11 and E30.

Overall, 28 different EV types belonging to three different species were identified. The predominant species was EV-B, with 122 isolates distributed among 18 types. Twenty-nine strains belonged to eight different types within species EV-A. Four isolates belonged to species EV-C, whereas no EV-D EV was identified.

Species B was detected throughout the 11-year period. Strains belonging to species A were first detected in 2011 and were then detected during the subsequent years. NPEVs belonging to species C were detected only in 2007, 2009 and 2014. 

### 3.2. Poliovirus Detection

Seventy-two samples, collected from 38 individuals, gave a cytopathic effect on the L20B cell line. All isolates were confirmed PV-positive with standard real-time RT-PCR tests using primers and probes specific to the three PV types: type 1 PV (*n* = 25), type 2 PV (*n* = 23) and type 3 PV (*n* = 24) (Table 3). The three types were detected over the study period except for type 2 PV which detection stopped in 2015. The PV isolation rate was 2.6%; a higher rate was obtained among patients with PIDs (6.8%), and lower rates were observed in AFP and healthy contacts (1.5% and 1.3%, respectively).

All L20B-positive samples were tested for their wild or Sabin origin. All isolates were identified as Sabin-related strains. Three individuals had a mixture of two PV types: PV1 + PV3 in two samples of two different PIDs patients and PV2 + PV3 in two samples from the same AFP patient. WPVs were not detected. 

The 72 Sabin-related PV strains were characterized by sequencing of the entire VP1 genomic region: 55 had 0 to 5 mutations and were characterized as Sabin-like, while 11 isolates had 6 to 14 nucleotide changes and were classified as VDPVs (Table 4). Six types one and three PV isolates had six to eight nucleotide changes and were identified as pre-VDPVs. According to the definitions of VDPV, during the study period, four VDPVs type 1, five VDPVs type 2 and two VDPVs type 3 were identified. Nine of them were isolated from patients with PIDs and were then classified as iVDPVs. Two strains (PV3-S011-AFP-TUN-17 and PV3-S335-AFP-TUN-16) were isolated from a patient initially classified as an AFP case; after the sequencing results, immunological investigations showed that this patient suffered from HLA class II deficiency and was then classified as PID.

Four strains of PV type 1 had nonsynonymous mutations, with amino acid substitution at position 90 (nt2749) of the VP1 region. Among these strains, three had mutations from Ile to Met, which led the amino acid at this point to reverse to Mahoney (the wild strain). One strain changed from Ile to Leu. The amino acid at this position was changed to Met from Ile in the process of attenuation from the Mahoney strain to Sabin strain. Two mutations at other positions of attenuation from the wild virus strain to Sabin strain led to substitutions of amino acids: positions 99 (nt2775) and 106 (nt2795) of the VP1 region. At position 99, three strains (PV1-S326-PIDs-TUN-09, PV1-S358-PIDs-TUN-09, PV1-S047-PIDs-TUN-10) had mutations from Lys to Arg and one strain (PV1-S200-PIDs-TUN-13) from Lys to Asn (Table 4). The amino acid at this position was changed to Arg from Lys in the process of attenuation from the Mahoney strain to the Sabin strain. At position 106, three strains had mutations from Thr to Ala. The amino acid at this position was changed to Thr from Ala in the process of attenuation from the Mahoney strain to Sabin strain.

Among type 2 VDPV isolates, four strains had one particular nucleotide mutation on the VP1 region that led to the change in amino acid: from Ile to Thr of the nt2909 mutation. This mutation occurred at the amino acid position 143, which relates to the decrease of neurovirulence of type 2 wild virus strain. Furthermore, three strains had mutations in nt 2540, which led to amino acid change Pro20leu; this mutation is not known to be relevant.

The two isolated type 3 VDPV virus strains had the same mutations: at position 2637 with an amino acid change from Ala to Val and position 2790 with an amino acid change from Met to Thr. These mutations did not match with any of the described epitopes or attenuation positions.

## 4. Discussion

This report gives an overview of polio and nonpolio enterovirus circulation in Tunisia over an 11-year period starting from 2007. 

Twenty-eight different types of nonpolio enteroviruses (NPEVs) were identified during the study period; the NPEV detection rate varied between 2.4% and 11.2% per year, with a mean rate of 5.8%. These results are similar to those published in a previous study conducted in Tunisia between 1992 and 2003, where 23 different types of NPEV were detected, with a detection rate ranging from 3.0 to 10.8 per year and a mean rate of 5.3% [15]. Thus, our results further confirm the relatively low circulation of NPEVs in Tunisia. Indeed, it is generally considered that in most developing countries, the annual NPEV detection rate exceeds 10% of stool samples assessed and may reach up to 50% in some countries [25]; however, it may also be less than 10% in other countries [26], depending on the socioeconomic level and climatic factors. According to the WHO standards, the annual NPEV isolation rate is used as an indicator to monitor the sensitivity of national PV surveillance programs and detect possible loss in the sensitivity of detection due to inadequate laboratory methods or to inadequate conditions for sample collection and transportation. Thus, it is important to document the NPEV isolation rate over years to help interpret this criterion.

In contrast, the distributions of the different NPEV species found in the present work were slightly different from the one described in a previous study between 1992 and 2003 [15]. Although a total absence of circulation of species D and a predominance of species B was found in this series, together with the previous one, we noticed the emergence of viruses from species A since 2011, which represented most of the detected NPEV types in 2016 and 2017. The emergence of species A could be explained by the political situation in the country after the “Arab spring” revolutions in 2011, which saw the inflow of many refugees from different African origins and coming from Libya. Two types from species C, CV-A24 and CV-A21, were also detected in 2007, 2009 and 2014 in healthy contacts. 

The predominance of species B among circulating NPEVs was reported in many countries in Africa, Asia and Europe [27,28,29,30,31]. In the present study, E6, E11, E14 and E30 were the most frequently isolated and seem to have an endemic and continuous circulation. E6 was the most prevalent EV overall and the most prevalent type in 2011. E6 is one of the most frequently identified EV types worldwide and usually associated with outbreaks [32,33,34,35,36,37]. An increase in E6 incidence was seen in the Netherlands in 2016 [38], and a high prevalence of E6 was also reported in many countries in Asia and Africa such as Cameroon, Nigeria, Eastern China, Pakistan and the Philippines [32,39,40]. E11 and E30 regularly cause large outbreaks worldwide. In Europe, E11 and E30 were the most frequently detected EV types [41,42,43]. Concerning E14, little is known about its epidemiology worldwide; it was rarely detected previously in Tunisia but was more frequent in the present series.

In contrast, other NPEV types were isolated much more rarely during our study period with very few isolates: CV-A2, CV-A6, EV-A76, E2, E4, E9, CV-A24. This suggests that they are not endemic and were just occasionally introduced in the country. Compared to previous studies conducted in Tunisia, EV-A71 is detected for the first time in Tunisia, at least in AFP cases and healthy contacts [15]. EV-A71 is one of the most important causative agents of hand, foot and mouth disease; however, EV-A71 can also cause a diverse range of neurological diseases, including brainstem encephalitis and neurogenic pulmonary edema and is now considered as a possible etiological agent for AFP [44].

This high diversity in NPEVs, the changes in their patterns of circulation, and the emergence of some types highlight the interest to maintain continuous surveillance of these pathogens, especially in susceptible populations.

In addition to NPEVs, PVs were also detected but all isolates had a Sabin origin with no Wild PV detected. This indicates that Tunisia is still maintaining its polio-free status despite the critical political situation since 2011, unlike other countries such as Syria, where WPVs were reintroduced due to political issues after a long PV-free period [45].

PVs of Sabin origin were detected during the study period with an overall detection rate of 2.6% of tested samples. This is quite expected in a country still using OPV in its national immunization program. In a previous study [15], the PV detection rate was 3.3% in AFP cases and their healthy contacts from 1992 to 1997, a period when frequent supplemental immunization campaigns with OPV were conducted in addition to routine immunization of neonates. The rate decreased from 1998 to 2003 when supplemental immunization campaigns with OPV stopped. If we consider AFP cases and their healthy contacts only, the PV detection rate found herein is further decreased (1.5% and 1.3%, respectively), and PV type 2 has not been detected since 2015 [15]. In fact, after the certification of WPV2 eradication, in September 2015, by the Global Commission for the Certification of Polio Eradication, almost all countries using trivalent OPV (tOPV), switched to bivalent OPV (bOPV), containing live attenuated poliovirus types 1 and 3 only [46]. Tunisia switched to bOPV in 2016. This, together with the replacement of two OPV doses by IPV in 2015 and 2016, led to the further decrease of the PV detection rates in AFP cases and their healthy contacts. The higher overall rate found in the present study (2,6%) is due to the presence of PIDs in the study population in whom most PV isolates were detected, the isolation rate in these patients being 6.8% (Table 3). The introduction of PID patients in the PV surveillance strategies, in addition to AFP cases, is now seriously considered as a supplemental strategy to enhance the sensitivity of PV detection, particularly in these advanced phases of the global polio eradication program, and our results support the usefulness of such strategies.

A total of 72 Sabin-like poliovirus strains were characterized by sequencing of the entire VP1 genomic region: 61 had 0 to 5 mutations for type 2 PV and 0 to 9 for types 1 and 3 PV and were characterized as Sabin-like, according to WHO standards. Among them, six strains of types 1 and 3 PV had between six and nine nucleotide changes, and they are considered high-mutant strains or pre-VDPV. Five type 2 PV had six mutations or more, four strains belonging to type 1 PV and two strains belonging to type 3 PV had 10 mutations or more. They were classified as VDPVs. Nine out of the eleven VDPVs identified were isolated from nonparalyzed PID patients and were classified as iVDPVs. The remaining two identified VDPVs were isolated from a patient initially classified as AFP case but, after sequencing results, immunological investigations showed that this patient suffers from HLA class II deficiency and were then classified as iVDPVs. 

In this work, we also studied the mutation profiles of VDPV strains in the entire VP1 gene and noticed recurrent mutations that occurred in VDPV types 1, 2 and 3, leading to nonsynonymous mutations. Three VDPVs of type 1 strains contained the reverse mutation of nt2795, which is known to increase the neurovirulence of Sabin strains [47]. This mutation was also detected in two pre-VDPV strains and was previously described among a vaccine-associated paralytic poliomyelitis (VAPP) case in China [48]. It is well known that PV type-1-attenuated live vaccines (Sabin strain) have acquired 57 nucleotide mutations and 21 amino acid substitutions in the process of attenuation from the neurovirulent parental Mahoney strain [49]. Nucleotide mutations that were known to cause the attenuation occurred at nt935 (VP4), nt2438 (VP3), nt2795 and nt2879 (VP1) [48]. With regard to VDPVs of type2 and through mouse experiments, Ren et al. demonstrated that the major attenuation position is amino acid position 143 in the VP1, from Thr (Lansing strain) or Val to Ile (Sabin strain) [50]. In this study, we found the reverse mutation (Ile to Thr) atnt2909 in4 VDPV type 2 isolates. 

Concerning VDPVs of type 3, two mutations were found at positions 2637 and 2790. The same mutations were also found in the four pre-VDPV. These mutations did not match with any described epitopes or attenuation positions.

These findings encourage systematic sequencing of the entire VP1 genomic region or further whole-genome sequencing for countries with a low poliovirus circulation to enhance the surveillance of these viruses. 

## 5. Conclusions

In conclusion, this study contributes to a better knowledge of circulating enteroviruses in Tunisia and the changes in their epidemiology with time. It may constitute a model to other countries that have eradicated polioviruses or are very close to eradication. It shows that keeping strong AFP surveillance and continuous monitoring of the excretion of EVs in PID patients is crucial to maintain polio-free status and rapidly detect any emerging polio or nonpolio enterovirus strain of special concern for human health.

## Figures and Tables

**Table 1:** Description of the studied population and stool sampling.

		Number of Individuals per Year (Stool Samples)										
Clinical Status	Total Studied Individuals (Stool Samples)	2007	2008	2009	2010	2011	2012	2013	2014	2015	2016	2017
Paralytic cases	607 (1296)	49 (109)	60 (129)	42 (85)	58 (126)	48 (101)	56 (123)	41 (86)	43 (91)	61 (126)	87 (184)	62 (136)
Healthy contacts	825 (825)	154 (154)	96 (96)	60 (60)	65 (65)	34 (34)	85 (85)	30 (30)	60 (60)	80 (80)	61 (61)	100 (100)
Immunodeficients	290 (614)	14 (22)	18 (36)	53 (118)	61 (138)	20 (61)	23 (50)	19 (37)	41 (83)	21 (35)	11 (20)	9 (14)
TOTAL	1722 (2735)	217 (285)	174 (261)	155 (263)	184 (329)	102 (196)	164 (258)	90 (153)	144 (234)	162 (241)	104 (265)	171 (250)

**Table 2:** Distribution of nonpolio enterovirus (NPEV) types identified per year.

Species	Types	2007	2008	2009	2010	2011	2012	2013	2014	2015	2016	2017	Total
A	**CV-A2**	0	0	0	0	0	**1**	0	0	0	**1**	0	**2**
	**CV-A4**	0	0	0	0	**3**	0	**1**	**1**	**1**	0	**2**	**8**
	**CV-A6**	0	0	0	0	0	0	0	0	0	**2**	0	**2**
	**CV-A8**	0	0	0	0	0	**1**	0	0	**2**	0	0	**3**
	**CV-A10**	0	0	0	0	0	0	0	0	0	**1**	**3**	**4**
	**CV-A16**	0	0	0	0	0	0	0	0	0	**3**	**2**	**5**
	**EV-A71**	0	0	0	0	0	0	0	**2**	0	**2**	0	**4**
	**EV-A76**	0	0	0	0	0	0	0	0	0	**1**	0	**1**
B	**CV-B2**	0	0	0	0	**3**	0	0	0	0	0	0	**3**
	**CV-B4**	0	0	0	0	0	**3**	0	0	0	0	**1**	**4**
	**CVB5**	0	0	**4**	0	0	0	0	0	0	0	0	**4**
	**E2**	0	0	0	0	0	0	0	**1**	0	0	0	**1**
	**E3**	**3**	**2**	0	0	0	0	0	0	**1**	0	0	**6**
	**E4**	0	0	0	0	**1**	0	0	0	0	0	0	**1**
	**E6**	0	0	**5**	**4**	**10**	0	0	0	**3**	0	0	**22**
	**E7**	0	0	0	0	1	0	0	0	0	**3**	**1**	**5**
	**E9**	0	0	0	0	0	**1**	0	0	0	0	0	**1**
	**E11**	**1**	**3**	0	**7**	0	0	**2**	0	0	0	**2**	**15**
	**E13**	0	0	0	**3**	0	0	0	**1**	0	0	0	**4**
	**E14**	0	0	**4**	**11**	0	0	0	0	0	3	0	**18**
	**E16**	0	0	**4**	0	0	0	0	0	0	0	0	**4**
	**E20**	0	0	0	0	0	**5**	0	0	0	0	0	**5**
	**E21**	**1**	**5**	0	0	0	0	0	**2**	0	0	0	**8**
	**E24**	0	**2**	0	0	0	0	0	0	0	0	0	**2**
	**E25**	0	0	0	0	**2**	**2**	0	0	**4**	0	0	**8**
	**E30**	0	0	0	**5**	**2**	**1**	0	**2**	0	0	**1**	**11**
C	**CV-A21**	**1**	0	0	0	0	0	0	**2**	0	0	0	**3**
	**CV-A24**	0	0	**1**	0	0	0	0	0	0	0	0	**1**
	**Total**	**6**	**12**	**18**	**30**	**22**	**14**	**3**	**11**	**11**	**16**	**12**	**155**
	**Positivity Rate**	2.1	4.6	6.8	9.1	11.2	5.4	2.0	4.7	4.6	6.0	4.8	

**Table 3:** Distribution of polioviruses identified per clinical status and per year.

	N° of Stool Samples	N° of Positive Polioviruses Per Clinical Status Per Year			Total N°	Positivity Rate
		PV1	PV2	PV3		
AFP *	**1296**	**7**	4	8	19	1.5
**Healthy contacts**	825	4	3	4	11	1.3
Patients with PIDs **	614	14	16	12	42	6.8
**Total**	**2735**	**25**	**23**	**24**	**72**	**2.6**
**2007**	285	0	6	0	6	2.1
**2008**	261	1	1	2	4	1.5
**2009**	263	5	3	2	10	3.8
**2010**	329	6	2	4	12	3.6
**2011**	196	0	2	1	3	1.5
**2012**	258	1	5	2	8	3.1
**2013**	153	5	0	0	5	3.3
**2014**	234	3	4	3	10	4.3
**2015**	241	1	0	3	4	1.7
**2016**	265	0	0	4	4	1.5
**2017**	250	3	0	3	6	2.4
**Total**	**2735**	**25**	**23**	**24**	**72**	**2.6**

* AFP= acute flaccid paralysis, ** PID = primary immunodeficiency.

**Table 4:** Nucleotide and amino acid changes in the entire VP1 gene of vaccine-derived polioviruses (VDPVs) identified in comparison with Sabin strains.

Serotype	Isolated Strains	N° of mutations in the Entire VP1 Region	Synonymous Mutations	Nonsynonymous Mutations	Amino Acid Changes	N° of Mixed Bases	Position(s) of Mixed Base(s)	Final N° of Nucleotide Changes
**Type 1**	PV1 *-S326-PIDs ^+^-TUN-09	5	C2722U, G2740A	G2749A, A2775G, A2795G	Ile90Met, Lys99Arg, Thr106Ala	7	C2544Y ^a^, G2701R ^b^, U2731W ^c^, C2752Y, U2872Y, C3088Y, G3376R	12
	PV1-S358-PIDs-TUN-09	14	G2678A, C2722U, G2740A, C2761U, U2767C, U2872C, U3154C, G3292A, G3371A, G3376A	C2544U, G2749A, A2775G, A2795G	Thr22Met, Ile90Met, Lys99Arg, Thr106Ala,	0		14
	PV1-S047-PIDs-TUN-10	11	U2569C, C2722U, G2740A, U2872C, C3144U, G3292A, G3376A	C2544U, G2749A, A2775G, A2795G	Thr22Met, Ile90Met, Lys99Arg, Thr106Ala,	0		11
	PV1-S200-PIDs-TUN-13	6	U2779C, C2968U, A3033U, U3352C	A2747U, G2776U	Ile90Leu, Lys99Asn	5	G2620R, U2626Y, G2957R, A3079Y, U3169Y	11
**Type 2**	PV2-S177-PIDs-TUN-14	9	G2502A, A2518G, C2560U, A2628G, A2718G, C2787U, A2992G, G3093A, U3111C			0		9
	PV2-S293-PIDs-TUN-07	3	G3048A	C2540U, U2909C	Pro20Leu, Ile143Thr	6	U2499Y, G2625R, C2706Y, A2838R, G2892R, A3375R	8
	PV2-S294-PIDs-TUN-07	3	G3048A	C2540U, U2909C	Pro20Leu, Ile143Thr	6	U2499Y, G2625R, C2706Y, A2838R, G2892R, A3375R	10
	PV2-S297-PIDs-TUN-07	5	U2523C, C2595U, G2694A, U2814A	U2909C	Ile143Thr	1	A3122R	6
	PV2-S001-PIDs-TUN-08	4	G2892A, G3048A	C2540U, U2909C	Pro20Leu, Ile143Thr	4	U2499Y, G2625R, C2706Y, A3375R	8
**Type 3**	PV3-S335-AFP ^|^-TUN-16	2		C2637U, U2790C	Ala54Val, Met105Thr	8	U2503W, A2716R, U2876Y, G2888K ^d^, A2897R, A3017R, G3107K, A3232R	10
	PV3-S011-AFP-TUN-17	10	U2860C, U3205A, U3364C	C2637U, U2790C, G2888U, A2897G, A3017G, A3111G, G3334U	Ala54Val, Met105Thr, Ala137Ser, Ala138Ser, Ile181Val, Lys212Arg, Arg286Ser	1	G2866R	11

^a^ Y = C or T, ^b^ R = A or G, ^c^ W = A or T, ^d^ K = G or T, ^|^ AFP = acute flaccid paralysis, ^+^ PID = primary immunodeficiencies, * PV1 = poliovirus type 1, PV2 = poliovirus type 2, PV3 = poliovirus type 3.

## Data Availability

Not applicable.
